# Studying phenological phenomena in subarctic biomes with international school pupils as citizen scientists

**DOI:** 10.1002/ece3.7122

**Published:** 2020-12-30

**Authors:** Cornelya F. C. Klütsch, Paul Eric Aspholm, Natalia Polikarpova, Olga Veisblium, Tor‐Arne Bjørn, Anne Wikan, Victoria Gonzalez, Snorre B. Hagen

**Affiliations:** ^1^ Norwegian Institute of Bioeconomy Research NIBIO – Division of Environment and Natural Resources Ås Norway; ^2^ Norwegian Institute of Bioeconomy Research NIBIO – Division of Forest and Forest Resources Ås Norway; ^3^ Pasvik State Nature Reserve Rayakosky Russia; ^4^ Kandalaksha State Nature Reserve Kandalaksha Russia

**Keywords:** climate change, environmental education, international collaboration, phenology, school‐based citizen science, STEM education, sustainability education, taiga, transformative learning, tundra

## Abstract

Citizen science can facilitate in‐depth learning for pupils and students, contribute to scientific research, and permit civic participation. Here, we describe the development of the transnational school‐based citizen science project *Phenology of the North Calotte*. Its primary goal is to introduce pupils (age 12–15; grades 7–10) in northern Norway, Russia, and Finland to the local and global challenges of climate change resulting in life cycle changes at different trophic and ecosystem levels in their backyards. Partnerships between regional scientists and staff from NIBIO Svanhovd, State nature reserves, national parks, and teachers and pupils from regional schools aim to engage pupils in project‐based learning. The project uses standardized protocols, translated into the different languages of participating schools. The phenological observations are centered around documenting clearly defined life cycle phases (e.g., first appearance of species, flowering, ripening, leaf yellowing, snow fall, and melt). The observations are collected either on paper and are subsequently submitted manually to an open‐source online database or submitted directly via a newly developed mobile app. In the long term, the database is anticipated to contribute to research studying changes in phenology at different trophic levels. In principle, guided school‐based citizen science projects have the potential to contribute to increased environmental awareness and education and thereby to transformative learning at the societal level while contributing to scientific progress of understudied biomes, like the northern taiga and (sub)arctic tundra. However, differences in school systems and funding insecurity for some schools have been major prohibiting factors for long‐term retention of pupils/schools in the program. Project‐based and multidisciplinary learning, although pedagogically desired, has been partially difficult to implement in participating schools, pointing to the need of structural changes in national school curricula and funding schemes as well as continuous offers for training and networking for teachers.

## Graphical abstract

The primary goal of the transnational school‐based citizen science project *Phenology of the North Calotte* is to introduce pupils (age 12–15) in northern Norway, Russia, and Finland to the local and global challenges of climate change resulting in life cycle changes at different trophic and ecosystem levels in their backyards. Pupils collect phenological observations either on paper and these are subsequently submitted manually to an open‐source online database or they are submitted directly via a newly developed mobile app. Continued school participation is dependent on reliable funding, training opportunities for teachers to implement project‐based citizen science projects in their schools, and structural changes in school curricula to provide more freedom for project‐based and multidisciplinary work.

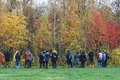

## 1 INTRODUCTION

**FIGURE 1:**
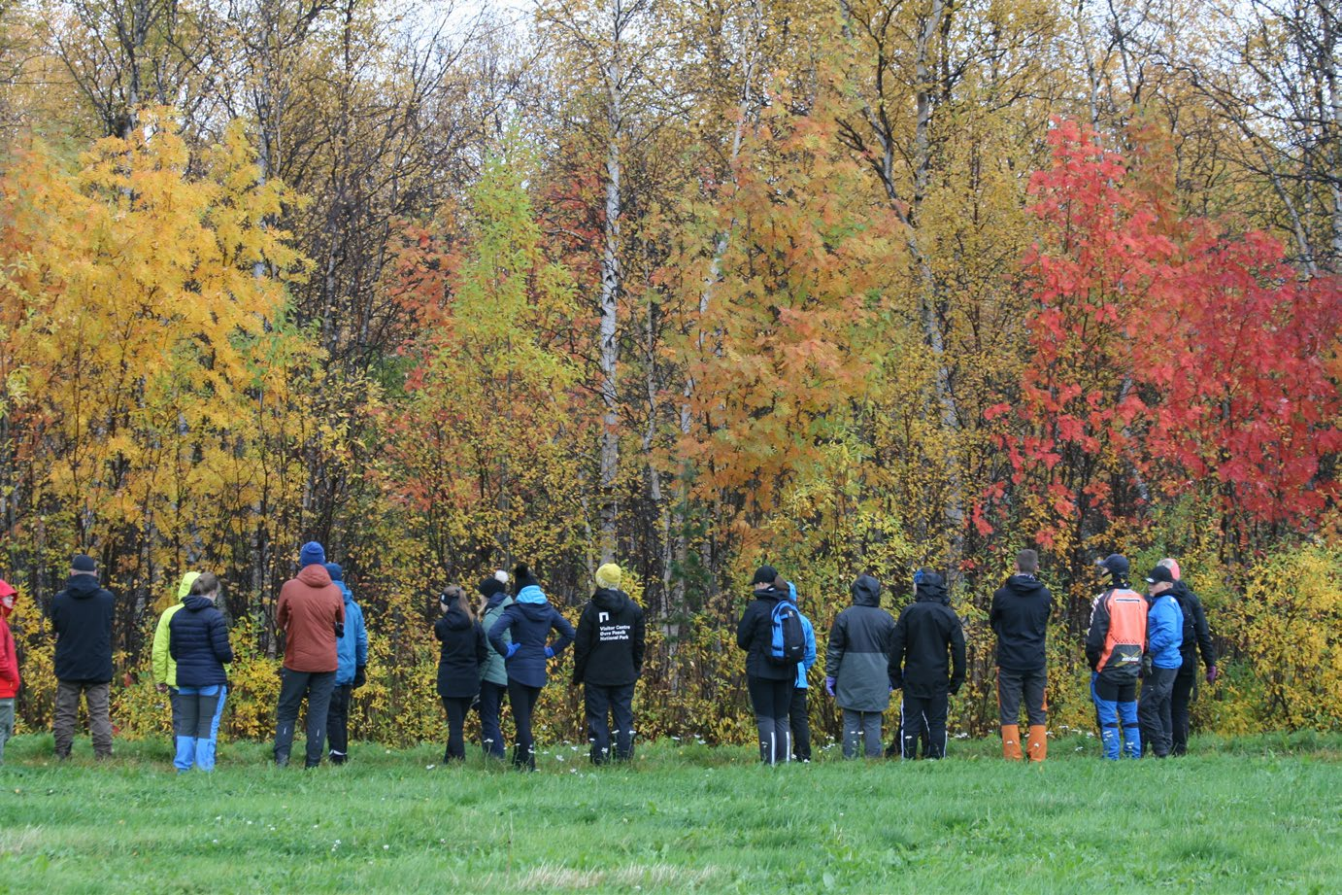
School pupils and their teachers on a field trip learning about the phenological changes in autumn (e.g., changing leaf colors) and the usage of the mobile app. Photo credit: Helena Klöckener (NIBIO Svanhovd)

Citizen science, broadly defined as the participation of members of the public in scientific research, contributes to scientific and environmental education for pupils and students and civic involvement in scientific research (DITOs Consortium, 2019; MacKenzie et al., 2017; Mayer, 2010; Turrini et al., 2018). Approaches for citizen science projects are diverse and range from, for instance, bioblitzes for species registrations and counts as well as long‐term ecological monitoring like phenological observations studying the impact of climate change on the timing of biological processes, to digital citizen science projects for the analyses of large data sets like photograph classifications as hosted, for example, by Zooniverse (https://www.zooniverse.org/; DITOs consortium, 2017; MacKenzie et al., 2017; Saunders et al., 2018). Although all citizen science projects contribute to scientific research to some extent, main goals may differ and are dependent on the target group of participants (Figure 1).

School‐based citizen science projects offer the opportunity to engage pupils in ecological research and contribute to both their scientific and environmental education (DITOs Consortium, 2019; Harris et al., 2020; Ruiz‐Mallén et al., 2016; Wals et al., 2014). Wals et al., (2014) proposed citizen science as a tool to combine scientific education, usually focussed on learning outcomes connected to knowledge and practical skill development, with environmental education, which considers values, attitudes, and changing behaviors, to create a holistic learning experience. Transformative learning, which includes the deconstruction of preconceived notions, knowledge, attitudes, and behavior to generate new shared knowledge, is central to environmental education that aims to lead to societal shifts toward sustainability (Cebrián & Junyent, 2015; Wals et al., 2014). Key competences for sustainability development include, for example, dealing with uncertainty, forward‐looking manner, open‐minded perception, trans‐cultural understanding and collaboration, the reflection of individual and cultural concepts, planning and implementation, the ability to feel empathy, sympathy, solidarity, and motivating self and others (Cebrián & Junyent, 2015). Further, for scientific and environmental education to be engaging and effective, it should be purposeful, goal‐oriented, intentional, and authentic (Saunders et al., 2018). Citizen science projects generally meet these requirements well, because citizen scientists, including schoolchildren, can participate and contribute to a larger scientific initiative with a clear scientific purpose in mind that tackles a real‐world problem and seeks social change through increased awareness that potentially promotes ecological stewardship (Wals et al., 2014).

Phenological observations of the annual timing of natural events in plant and animal species are ideal topics for (school‐based) citizen science projects because they are usually comparatively easy to record and require relatively little training (Mayer, 2010). Phenological observations include, for example, the first occurrence of migratory and overwintering animal species or the first flowering of plant species (Table 1). Not surprisingly, numerous citizen science projects using phenological observations have been established in recent years (e.g., Hulbert et al., 2019; Kosmala et al., 2016; MacKenzie et al., 2017; Mayer, 2010). In high‐latitude environments, like subarctic tundra and northern taiga, severe shifts have been observed in phenological timelines (Prevéy et al., 2017). This coincides with the highest level of global warming, which is 2–3 × higher in (sub)arctic regions than the global average, and it is expected that these regions will continue to experience a considerable increase in average annual temperatures (Anderegg & Diffenbaugh, 2015; Post et al., 2009). Hence, future changes in phenology are anticipated to be most drastic in high‐latitude environments (Prevéy et al., 2017), potentially leading to mismatches in the timing of life‐history events of interacting species that affect their performance like trophic interactions, prey–predator relationships, and pollinator–plant interactions (Chmura et al., 2019; Damien & Tugeron, 2019; Renner & Zohner, 2018), reproductive events (Wann et al., 2019), demography (Miller‐Rushing et al., 2010; Visser & Gienapp, 2019), migration events (Koleček et al., 2020), and ecosystem functions like nutrient cycling (Beard et al., 2019; Leffler et al., 2019). In addition, range expansions of species due to climate change will alter the species compositions of (sub)arctic and boreal ecosystems (Macgregor et al., 2019).

**TABLE 1:** Selected species representing different trophic levels in the tundra and taiga biomes and their respective ecosystem services

Common name	Latin name	Phenophases	Ecosystem services
Berries and flowers			
Globe flower	*Trollius europaeus*	Flowering	
Bilberry	*Vaccinum myrtillus*	Flowering	Provisioning service: sweet flowers for tea and medicine Regulating service: food for other animals
Bilberry		Ripening	Provisioning service: food Regulating service: food for other animals Cultural service: collecting berries together—spending time with friends or family Learning how to collect berries
Labrador tea	*Rhododendron palustre*	Flowering	Provisioning service: flowers and leaves for tea
Rosebay willowherb	*Epilobium angustifolium*	Flowering	Provisioning service: flowers and leaves for tea
Rosebay willowherb		Spreading of seeds	Provisioning service: seed wool for medicine
Cloudberry	*Rubus charmaemorus*	Flowering	Provisioning service: flowers for tea and medicine
Cloudberry		Ripening	Provisioning service: food
Dwarf cornel	*Chamaepericly‐menum suecica*	Flowering	
Dwarf cornel		Ripening	
Cowberry	*Vaccinium vitis‐idaea*	Flowering	Provisioning service: flowers for tea and medicine
Cowberry		Ripening	Provisioning service: food and medicine
Tree species			
Downy birch	*Betula pubescens*	Leaves unrolling	Provisioning service: young leaves for tea and medicine, indicator of time to collect sap, production of soap
Downy birch		Flowering	Indicate provisioning service: collect branches for sauna sweepers
Downy birch		Becoming yellow (50%)	Regulating service: influence on soil
Downy birch		Start leaves falling	Indication provisioning service: cutting wood for fire
Rowan	*Sorbus aucuparia*	First green leaves	Regulating service: growth of tree begins—CO_2_ storage
Rowan		Flowering	Regulating service: pollination provisioning service: flowers for medicine and tea
Rowan		Becoming yellow (50%)	Regulating service: growth stops
Rowan		Ripening	Provisioning service: food Regulating service: attract birds that earlier were hunted
Rowan		Start falling leaves	Provisioning service: wood products
Insect species			
Mosquito	Cullicidae	First bite	Regulating services: food for other animals influence on our immune‐system
Ant	*Formica*	First ants on the hill	
Bumble‐bee	*Bombus*	First observation	Regulating services: pollination starts
Bird species			
Bluethroat	*Luscinia svecica*	First observation	Regulating services: eats and fertilizes Cultural service: recreation and nature‐based tourism (i.e., bird‐watching) Earlier provisioning service: food Cultural services: signs and mythology—beliefs
Common cuckoo	*Cuculus canorus*	First observation	Regulating services: eats and fertilizes Cultural service: recreation and nature‐based tourism Earlier provisioning service: food Cultural services: signs and mythology—beliefs
White wagtail	*Motacilla alba*	First observation	Regulating services: eats and fertilizes Cultural service: recreation and nature‐based tourism Earlier provisioning service: food Cultural services: signs and mythology—beliefs
Willow warbler	*Phylloscopus trochilus*	First observation	Regulating services: eats and fertilizes Cultural service: recreation and nature‐based tourism Earlier provisioning service: food Cultural services: signs and mythology—beliefs
Arctic tern	*Sterna paradisaea*	First observation	Regulating services: eats and fertilizes Cultural service: recreation and nature‐based tourism Earlier provisioning service: food Cultural services: signs and mythology—beliefs
Snow bunting	*Plectrophenax nivalis*	First observation	Regulating services: eats and fertilizes Cultural service: recreation and nature‐based tourism Earlier provisioning service: food Cultural services: signs and mythology—beliefs
Meteorological parameters			
Snow		First snow laying	
Snow		Fields free from snow	
Ice		Waters covered by ice	
Ice		Waters free of ice	

Note

All species with common and scientific names, together with the different phenophases, are listed. Insects are not identified to the species level as this would be too difficult to do. For ecosystem services, information is only listed if this is taught in the project. Hence, not all ecosystem services are listed and the reason for this has mainly been time constraints in lesson plans.

However, regardless of increasing evidence that fundamental phenological changes will occur in (sub)arctic tundra and taiga biomes, a recent literature research found that high‐latitude environments are the least researched and that the proportion of research dedicated to, for example, the tundra decreased between 2000 and 2015 (Diepstraten et al., 2018). Potential causes for this trend are limitations in access to remote areas coupled with a comparatively low human population density, high access costs, and high complexity of interactions studied as well as the need for long‐term monitoring (Diepstraten et al., 2018). This creates a vacuum for long‐term monitoring efforts, which are essential to study environmental, ecological, and evolutionary changes due to climatic and seasonal changes. Citizen science projects increasingly fill a gap for research and environmental monitoring where repeated observations are needed for large geographical areas that scientists alone cannot cover (Mayer, 2010; McKinley et al., 2017; Miller‐Rushing et al., 2012).

Here, we present the design and implementation of a school‐based citizen science project attempting to bridge the gap between science and environmental education toward an interdisciplinary transformative learning experience while contributing to phenology research in high‐latitude ecosystems (Ruiz‐Mallén et al., 2016; Wals et al., 2014). The main goal of this article is to use the *Phenology of the North Calotte* school‐based citizen science project as a case study to illustrate some of the main project components and how they link to certain learning outcomes for scientific and environmental education. By doing so, we provide some guidelines for others that are interested in building up similar school‐based citizen science projects. Further, we explain in detail how key project elements (e.g., quality assurance) were implemented with reference to recent literature to highlight potential pitfalls and solutions.

The main goals of this project are as follows:

1. Educate pupils on climate and environmental change and scientific methods, including the use of Internet and mobile applications for gathering observations, as well as provide teachers with assistance in project‐oriented pedagogy and environmental education.
2. Build a network of citizen science observers in a transnational setting to develop a local and regional network of actors for long‐term environmental stewardship.
3. Provide long‐term data resources for local and regional researchers, natural resource and conservation managers, and decision‐makers to track environmental changes associated with climate change.

## 2 HISTORY, BACKGROUND, AND SOME OUTCOMES OF THE PROJECT

**FIGURE 2:**
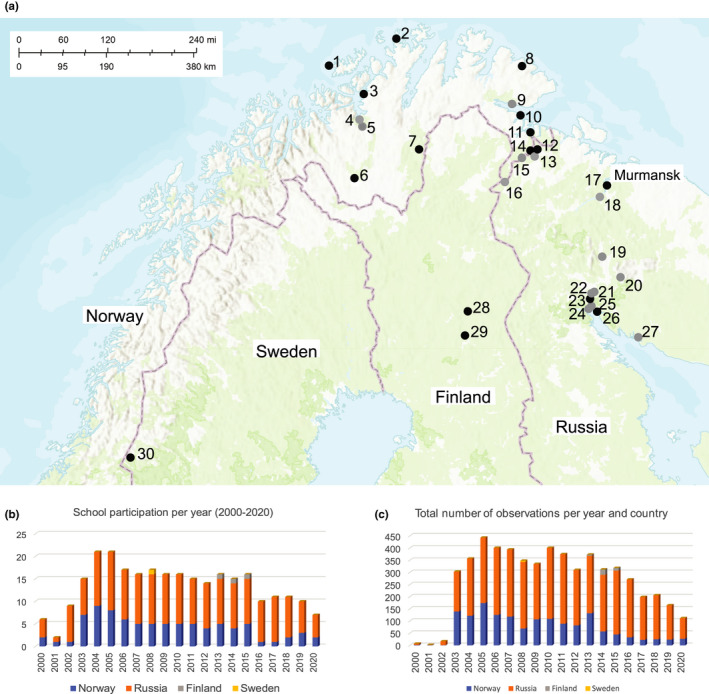
Overview about the study area and some project statistics. (a) shows the geographical distribution of all contributing schools; in light gray, schools and educational youth centers that have participated and registered data for more than 10 years in the project are highlighted. The numbers indicate the different schools participating: 1 = Sorvaer Oppvekstsenter (Norway), 2 = Havoysund school (Norway), 3 = Korsfjord, Oppvekstsenter (Norway), 4 = Alta junior high school (Norway), 5 = Alta oppvekstkontor (Norway), 6 = Sámi University of Applied Sciences, Kautokeino (Norway), 7 = Karasjok primary school (Norway), 8 = Batsfjord school (Norway), 9 = Vestre Jakobselv school, 10 = Bugoynes Oppvekstsenter (Norway), 11 = Kirkenes junior high school (Norway), 12 = School 20, Nikel (Russia), 13 = School 1, Nikel (Russia), 14 = Skogfoss school, 15 = Pasvik school (Norway), 16 = School 11, Rajakoski (Russia), 17 = School 45, Murmansk (Russia), 18 = School 1, Murmashi (Russia), 19 = School 8, Monchegorsk (Russia), 20 = School 7, Bolshoy Vudiyavr, Kirovsk (Russia), 21 = Gymnasium 1, Poljarnie Zori (Russia), 22 = The Children's Creation Center, Poljarnie Zori (Russia), 23 = Africanda school 1, Poljarnie Zori (Russia), 24 = Children's ecological‐biological station, Kandalaksja (Russia), 25 = School 2, Kandalaksja (Russia), 26 = School 20, Luvenga (Russia), 27 = School 4, Umba Terskyi (Russia), 28 = Pelkoseniemi school (Finland), 29 = Hillatien school, Kemijärvi (Finland), 30 = Jormskolan, Jormvattnet (Sweden). Figure 2b displays the annual participation of Norwegian, Russian, Finnish (and Swedish) schools during the project period. Note that the numbers for 2020 are preliminary because registration was ongoing at the time the paper was written. (c) shows the annual number of phenological observations throughout the project duration. All figures are based on database entries and therefore do not include schools that participated in the training, but did not register data

The citizen science project *Phenology of the North Calotte* originated as an idea for a school‐based phenology network by Kandalaksha State Nature Reserve (Murmansk region and Republic of Karelia in Russia) and NIBIO Svanhovd (Sør‐Varanger commune, Finnmark county, Norwegian side of Pasvik river valley at the border with Russia), with both playing an active role in developing the program. Pasvik State Nature Reserve (Murmansk region, Russian side of transboundary Pasvik river valley) acted as a consulting role in this project from the beginning. The official citizen science project started in 2000 and was designed based on the national school curricula in Norway and Russia. It addresses mostly pupils between the ages of 12–15 years and their teachers. The program was an integrated part of the Barents Local Agenda 21 project at NIBIO Svanhovd. This initiative sought to increase the ecological knowledge and environmental awareness among pupils and teachers in Murmansk Oblast (Russia) and Finnmark County (Norway), although some schools from Northern Lapland (Finland) became associated with the initiative in 2008. The Environmental Education Network in Norway is responsible for the project's website and database (https://www.miljolare.no/en/aktiviteter/pnc/?nmlpreflang=en). Over the last 20 years, 26 schools, two youth centers, and one Sámi university (i.e., indigenous university in the Sámi territory in northern Norway) from the three countries have participated and registered observations in the online database (plus one school from Sweden for a year; Figure 2a), but participation of some schools varied over the years (Figure 2b). Namely, number of participating schools (Figure 2b) and data registration (Figure 2c) declined over time for Norway, while for Russia, participation and data registration remained comparatively stable. Currently, 12 schools and two youth centers (10 Russian and 4 Norwegian) are regularly participating in school gatherings and/or data registration and have been involved in these activities for at least 10 years (Figure 2a,b). School gatherings have exposed approx. 800–1,000 pupils to direct training from scientists and staff of the biological station NIBIO Svanhovd, state nature reserves, and national parks. The school‐based project work has reached more than 20,000 pupils so far.

The online database currently holds > 5,600 observations. The number of registrations per country and year is shown in Figure 2c. Mirroring the decline of number of schools in Norway participating in the program, the number of registered observations has been declining in recent years as well. Note that for 2020, the number of observations is preliminary as registration of observations was ongoing at the time when the article was written.

## 3 SPECIES AND METEOROLOGICAL PARAMETERS

Phenological observations preferably encompass a wide range of species and meteorological parameters that are typical for the ecosystem(s) studied and that allow for species interactions to be assessed. Northern ecosystems are generally dominated by long‐lived, slow‐growing plant species, which have adapted to harsh winters and short growing seasons. Similarly, animal species are adapted to short breeding seasons. Thus, even small changes in precipitation and temperature can be documented through phenological registrations in the most common northern species. In addition, the border region of Norway, Russia, and Finland is characterized by a series of different northern biomes, including the taiga, which is characterized by spruce, pine, birch, and larch forest. Further north, the tundra biome is found, dominated by dwarf shrubs, sedges, grasses, mosses, and lichens. Moreover, the eastern Siberian taiga meets the western boreal forest in the region, and therefore, it is particularly species‐rich. Monitoring these different environments long‐term allows for the comparison of phenological changes across microclimatic habitats.

However, species and associated phenophases must be easy to identify to ensure that reliable data are retrieved, especially when working with pupils. To meet both requirements, we have selected plant and animal species as well as meteorological parameters listed in Table 1. Species selection has been based on the following criteria: (a) ease of species identification and phase determination, (b) common occurrence in northern tundra and taiga biomes, (c) wide geographical distribution, (d) representative of ecological interactions and trophic levels (e.g., plant species plus pollinator of plant species, which serves also as food for bird species), and (e) ecosystem services provided by chosen species and meteorological parameters. The last criterion stresses that each species serves a purpose in the ecosystem. Further, by picking plant species that have been largely used by the local community for tea, medicine, and food, the importance of observed species for humans is underscored and pupils develop a better understanding of the dependency of humans on regional ecosystems. Pupils learn about different ecosystem service types like provisioning, regulating/maintaining, and cultural ecosystem services (see also Table 1). Importantly, former ecosystem services provided by some of the observed species are also touched upon to emphasize that systems are ever‐changing (i.e., temporal changes), offering the opportunity to introduce pupils to history and historical uses of their local environment.

Finally, meteorological parameters include ice and snow. These have been chosen because snow and ice cover duration are anticipated to decrease, and snow cover duration impacts the growing season for terrestrial plant species and breeding seasons for animals in high‐latitude environments (Derksen et al., 2015). For freshwater systems, earlier snow and ice melts can lead to mismatches for species that emerge from winter dormancy earlier, while food availability is low (Bokhorst et al., 2016). Furthermore, snowmelt in spring leads to a high influx of organic matter from land into water that influences nutrient cycling and food web dynamics (Bokhorst et al., 2016). Therefore, snow and ice phenology are clearly linked to ecosystem functions, which are often neglected in phenological studies (Beard et al., 2019).

## 4 TEACHER AND PUPIL TRAINING, PUPIL ENGAGEMENT, AND QUALITY CONTROL

The main challenges associated with citizen science projects are associated with data quality and standardization as the data retrieved needs to be of comparable quality to data obtained by professional scientists to be usable and seamless (Kosmala et al., 2016; Mayer, 2010). Recent studies proposed that schoolchildren can contribute to environmental and ecological research if research protocols are kept simple and if guidance by adults is provided (Makuch & Aczel, 2018; Saunders et al., 2018). Importantly, Castagneyrol et al., (2020) demonstrated that both schoolchildren and untrained adult scientists provide less reliable data in citizen science projects than professional scientists trained for the task at hand, highlighting the importance of providing adequate training and experience, but also that schoolchildren do not necessarily score worse than untrained adults. Working in groups might decrease variability in observational skills and make observations more reliable (Castagneyrol et al., 2020). Results by Ratnieks et al., (2016) suggest that it is important to have face‐to‐face training sessions of participants (both teachers and pupils). Further, re‐assessment of findings via pictures is recommended (Ekholm et al., 2019), particularly in a large multipartner setting to spot systematic biases and errors.

To meet these requirements, annual training for school teachers and subgroups of pupils from each school is provided with a teacher seminar in spring and pupil workshop in autumn with the latter alternating between Russian and Norway to foster cross‐boundary collaboration and cultural understanding among pupils. Scientists and educators from NIBIO Svanhovd, Russian state nature reserves, and specially invited speakers from other institutions are present in person and provide training concerning scientific background and field guides and clarify any upcoming issues. In addition, every year there is a special topic for participating teachers and pupils (e.g., plastic pollution, river ecosystems) to expand teaching materials and provide continuous education for both teachers and pupils. The topics are first presented to teachers in spring so that they have time to learn and prepare themselves for the pupil workshop in autumn. These training sessions have also been used to receive feedback from teachers on how to improve field guides to improve their clarity in the early stages of the project. In addition, teachers are provided with background knowledge of species, taxonomic identifications, ecosystem services, and links to local and historical knowledge. Camps also offer opportunities for teachers to experience field‐based instruction to enable them to subsequently teach pupils in their schools (Figure 1). The main aim of these training sessions is to empower teachers to implement a project‐based citizen science project embedded in their school curriculum and encourage networking between teachers from different schools. Finally, teachers can consult with scientists over phone, Skype, or email several times per year to discuss and solve upcoming problems.

As a response to the coronavirus crisis in 2020, the autumn meeting was conducted at NIBIO Svanhovd with Norwegian pupils attending in person, with social distancing and disinfection measures in place (e.g., school groups were seated socially distanced, hand washing/disinfection several times a day—particularly when entering common areas like cafeteria). Russian participants joined the meeting virtually via a streaming service because of travel restrictions. In addition, deliberations on how to use the website more effectively were conducted with the conclusion that future work should include preparing small informative videos about the species with their characteristics and phenology. In addition, short technical videos on mobile app usage may be useful as well.

**FIGURE 3:**
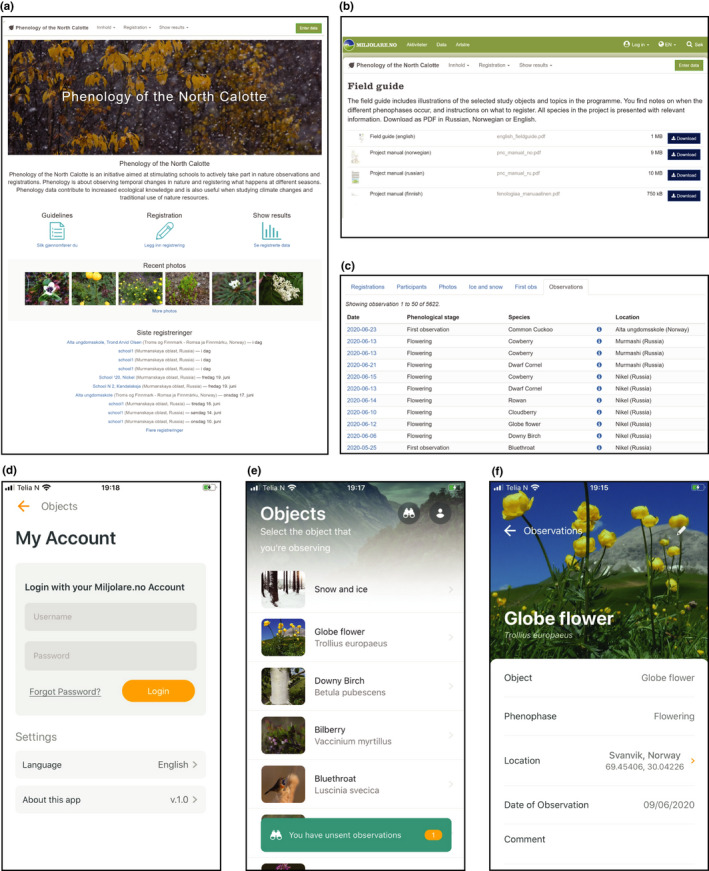
Screenshots from the website (a–c; https://www.miljolare.no/en/aktiviteter/pnc/?nmlpreflang=en) and the mobile app (d–f) for the *Phenology of the North Calotte* school‐based citizen science project. (a) Entry portal to the database with links to guidelines, registration, and results panel as well as the most recently submitted photographs and observations. (b) Webpage with downloadable, multilingual, field guides. (c) A snapshot of some submitted observations. (d) Registration/log‐in page of mobile app. (e) The objects page from which the species or meteorological parameters can be selected. Note that at the bottom of the figure, it says that there is one unsent observation. This notification reminds the observer to submit their observations that can first be stored locally on the mobile phone. (f) An example of a species page with the selected phenophases, georeferences, date, and comment field

In schools, the citizen science project is then built into the curriculum as a continuous, 2‐ to 3‐year project‐based learning tool for pupils (see Table 2 for learning outcomes related to this activity). Importantly, the long‐term involvement of pupils allows for reflection on lessons learned, worldviews, and personal beliefs. As it accompanies pupils during their personal development, it offers the opportunity of re‐engagement and transformative learning (Ruiz‐Mallén et al., 2016). As part of this project, pupils are asked to develop a “phenological path”. This includes the identification of a location near their school and/or neighborhoods where all species can be found or are at least passing by (e.g., bird migrations), and meteorological parameters can be observed. Pupils can consult with teachers during or after data collection to ensure that misunderstandings and misidentifications can be corrected as soon as possible. They usually work in small groups allowing them to learn from each other and discuss observations within groups to reduce error rates. Pupils upload their observations to an open‐source website: https://www.miljolare.no/en/aktiviteter/pnc/?nmlpreflang=en (Figure 3a–c). Data can be transformed to Excel worksheets, where further statistical analyses can be carried out. The website provides background information in form of field guides in several languages (i.e., English, Norwegian, Russian, and Finnish) that explain the phenophases (Table 1, Figure 3b) and species identification including photographs as well as tutorials for online technical support. The field guide with its parameters is based on principles of phenological observations used by the Russian state nature reserves and national parks that have a long‐standing tradition in recording phenological observations in the region. For example, Pasvik State Nature Reserve published a phenological guide—Atlas of plant’s phenophases (Polikarpova & Makarova, 2016). Finally, pupils are asked to submit photographs of their observations whenever possible to further verify their observations.

**TABLE 2:** Summary of different competence dimensions as outlined in Cebrián & Junyent (2015) and learning outcomes that the school‐based citizen science project *Phenology of the North Calotte* covers

Competences	Learning outcomes	Key points of project
Knowledge		
Introduction to some animal and bird species plus basic meteorological parameters	Knowledge of the natural environment	Pupils are taught morphological characteristics and names of typical tundra and taiga plant and animal species as well the functions of snow and ice in high‐latitude environments
Introduction to usages of the environment (agricultural, industrial, recreational) and pollution of the environment	Knowledge of the environment and environmental issues	Introduction to pollution sources in their environment like local and regional industries, urbanization, etc.
Knowledge about interactions of natural environment with social, cultural, and economic factors	Knowledge about benefits, functions, and importance of tundra and taiga ecosystems. Understanding socio‐economic dimensions	Discussion about competing interests in society, like economic growth and environmental protection. The importance of local industries to provide jobs
Language skills	Improvement of language skills	Some material is in English and therefore, language skills are trained. In meetings, English is often used as the common language
Practical skills		
Planning and implementation	Practical experience in scientific study design and implementation	Pupils need to identify locations where all species and meteorological parameters can be observed. Part of this action item are discussions, in which pupils must rationalize their location preferences. This mimics the process scientists go through while planning a study
Identification of natural diversity	Scientific classification of natural diversity	Pupils are taught morphological characteristics and names of typical tundra and taiga plant and animal species as well the functions of snow and ice in high‐latitude ecosystems
Ability to act	Knowledge about environmentally friendly actions to protect high‐latitude ecosystems	Education about waste reduction, litter collection initiatives, recycling, reduction of emissions (walking/bicycling to school), buying local products
Scientific work techniques	Knowledge about how scientists collect scientific data, understanding the scientific method, collaborative working, formulating conclusions from the observations/data Understanding of the knowledge‐building process	Introduction to phenophases that are used by professionals, like Russian state nature reserves, to observe timing of life cycles of different species in nature. Explanations how long‐term observation/monitoring can help science to understand changing environments due to climate change. Pupils are asked to work in small groups to discuss observations Example questions to be discussed: Which species have been found and what are possible reasons why certain species have not been found (in some regions)? Are there regional differences in phenology in different species and why if so?
Data management	Collection and compilation of data points; principles of reliable data acquisition and quality control	They take information with a mobile app and/or put data in an online database. Errors are discussed in class to emphasize the importance of attention to detail and accuracy of scientific data
Communication skills	Pupils learn how to present and discuss the results of their observations in writing and orally Improvement of language skills	Organization of seminars where pupils present their findings; sometimes internationally
Problem‐solving	Knowledge about potential solutions to environmental problems	Analysis and discussion about potential solutions to climate change and potential adaptation measures
Team work	Ability to work with others in a collaborative fashion	Group‐work in class, writing group essays, presenting as a group
Ethical values		
Environmental awareness	Awareness of the importance of high‐latitude environments and the species that live there	By introducing pupils to their direct backyard environment and the species living there. The value of a life (i.e., of a tree) by practical thinking
Individual and collective responsibility	Knowledge about how everyone can contribute to scientific research and that collaboration at different scales is needed for environmental protection of high‐latitude ecosystems	The project teaches pupils that other pupils from adjacent countries contribute to the same goal and face similar challenges. Similarly, protection of tundra and taiga ecosystems requires local, regional, and international action
Attitudes		
Respect for environment	Learn the value of natural environments	Teaching pupils what is special about the environment (in this case, tundra and taiga) they are living in. For example, what is the value (nonmonetary) of the species and the ecosystem functions by comparing functions.
Caring for environment	Knowledge how pupils can contribute to environmental protection	Education about waste reduction, litter collection initiatives, recycling, reduction of emissions (walking/bicycling to school), buying local products. Learning to envision different future scenarios and what will happen with the species and ecosystems. Understanding significance of scenarios by consequence analyses
Commitment, involvement, and active participation	Long‐term care and commitment to environmental protection and sustainable development	Pupils and teachers are part of a project that serves a larger purpose by being participants in this citizen science project. Why is it important that you engage in your neighborhood? Listing to what are the pros and cons
Coexisting, living and sharing experiences	Interaction and connection with different actors Collaborative working/team work and benefiting from the experience of others Practicing inclusivity and patience while working with others	Interacting and connecting with pupils within schools and from different schools
Emotions		
Sense of belonging to local environment	Place‐based connectivity	By making a connection to their immediate environment and the realization that they are dependent on the natural environment
Preparedness, outlook	Coping with uncertainty	By learning about climate change and the potential changes that will occur and have occurred in the past, pupils will feel better prepared facing uncertainty and changing environments

Note

Some of the learning outcomes and key elements of delivery are mutually nonexclusive, but rather overlapping and/or complementary. Details can differ between schools depending on national curricula.

## 5 MOBILE APP

The advancement of technology, including Internet enabling storage of data in online databases and mobile phones with cameras and GPS capabilities, has greatly contributed to the ease of data recording and the transfer of collected data into usable data sets (Mayer, 2010). A newly developed mobile app, available within the next few months for Apple products (implementations for other operational systems are planned), expands the technological aspect of the project. The main motivation to develop this app has been to avoid hand‐written notes as transcriptional errors can occur while taking GPS locations and dates, and while manually entering data into the online database, which also saves time. The app provides an interface that contains background information for each species including photographs of the species and description of phenophases to make this information readily available when needed (Figure 3d–f for some examples). In addition, citizen scientists can take photographs with their mobile phones/devices and submit those together with recorded observations. For each species, the different phenophases can be selected from a dropdown menu and the app can automatically determine geographical location (i.e., geographical coordinates) and presets the actual date to easily complement the record and avoid mistakes. A comment field lets observers add additional information, for instance, if there is doubt about an observation like uncertainty about the correct phenophases or species, which then can be checked by a scientist and/or teacher based on the submitted photograph and either corrected or discarded. By sending the observations, the data are automatically deposited into the online database (https://www.miljolare.no/en/aktiviteter/pnc/?nmlpreflang=en). The app will first be available in English and Russian, but further translations are planned. Currently, the app is free of charge, though so far only open to participating schools that have received training, which further ensures high‐quality data. However, inclusion of other societal groups is possible for future expansion of the project. Finally, to guarantee that data collection is possible while offline, as signal strength can greatly vary in the region, the observations can first be saved on the device to be sent later (i.e., online and offline functionality). Importantly, observations on paper with subsequent manual submission to the online database will remain possible as well to ensure that pupils, that do not own a mobile phone, can participate regardless in this activity.

## 6 LEARNING OUTCOMES

The United Nations (UN) Sustainable Development Goals include the provision of quality education, reduction of inequality, and gender equality (United Nations Department of Public Information, 2019). The here described project contributes to quality education by systematically addressing sustainable learning outcomes, combining scientific and environmental educational aspects into a school‐based citizen science project (summarized in Table 2). It supports the reduction of inequality and gender equality by including schools from different countries to provide transnational access to these educational resources. The aim is that schoolchildren of all genders from different school systems within countries (i.e., different types of secondary schools) and among countries (i.e., different national school curricula) regardless of socio‐economic background have access to sustainable development education. Further, by employing a project‐based framework, learning‐by‐doing and hands‐on learning is encouraged, which provides learning opportunities particularly for pupils that are practical learners and that struggle with classical learning techniques.

Improved sustainable development education at a young age encourages continued environmental stewardship and citizenship in adulthood (Makuch & Aczel, 2018). In addition, both teachers and pupils communicate to a certain degree to family members and friends, thereby adding to informal education in society. School‐based citizen science projects can either be built into the curriculum or they can be extracurricular activities. The former probably has a larger impact on pupils’ learning outcomes (Saunders et al., 2018), reaching a larger audience as many pupils of participating schools will be exposed to the activities during their school career. Sustainability for citizen science projects may be higher if they are built into the curriculum because continuous recruitment of new pupils is possible.

In this project, we have gone a step further with the inclusion of numerous schools in the region to generate connections at the local and regional level to establish a transnational network for sustainable development education. This network has three main purposes: (a) creating a sense of shared responsibility among schoolchildren from different schools and promote trans‐cultural understanding, (b) provide pupils with different spatial and temporal dimensions of their observations from their own backyard to local, regional, and global real‐world challenges and future changes, and (c) covering a larger area for observations. By involving regional schools, a network of local actors is developed that can support local and regional environmental stewardship in the long term. By observations of phenological phenomena, pupils have direct experiences with nature and their close surroundings, which has been acknowledged to be an essential tool to support environmental attitudes (Groulx et al., 2017; Schönfelder & Bogner, 2017).

With this approach, pupils are also introduced to systemic thinking that challenges them to understand interconnections of, for example, different spatial and temporal dimensions, cultures, societies, and socio‐economic developments. This makes them appreciate the complexities of systems and situations in sustainable development. These aspects have been identified as central to sustainable development education and the convergence of science and environmental education (Cebrián & Junyent, 2015; Wals et al., 2014). In this specific project, this increases awareness that climate change and linked environmental changes are borderless and that international collaboration is an important part of tackling climate change.

## 7 LESSONS LEARNED, REMAINING CHALLENGES, AND FUTURE DIRECTIONS

The implementation of this project has been largely successful and runs now for 20 consecutive years. Not surprisingly, however, some challenges remain in the implementation and maintenance in a comprehensive school‐based citizen science project like this one. The predominant problem is the long‐term retention of schools participating in this project. For example, the involvement of Norwegian and Finnish schools has decreased over the years. Reasons for school dropouts are manifold and can be summarized as follows:

- *Different national school curricula and project implementation among schools*: Different school systems with a range of pedagogical methods in the participating countries have led to difficulties in maintaining engagement and implementation of project‐based learning. In addition, different schools approach their national curriculum in different ways, leading sometimes to difficulties implementing the project. Adding to this is the fact that national curricula are usually dense, which results in shallow coverage of a diverse range of topics (Krajcik et al., 2007). This leaves not much room for project‐oriented work, especially if it is intended to span over several school years. A related issue is that there is no coordination of educational departments between countries and therefore, organization only occurs at the project level.
- *Variability in teacher interests and turnover of teachers*: Some schools dropped out because there was a change in the responsible teacher (e.g., teacher retired or moved to another school) with the new teacher having less interest in continuing the project. Further, personal interests of teachers differed due to, for instance, their subject combination they teach, and biology was not their main subject. The teacher turnover also leads to higher workload for scientists and staff from research institutions and state nature reserves/national parks because they must actively follow up with schools every year to ensure continued engagement from schools. Sometimes, the school management changed, and this project was deemed not relevant anymore under new leadership.
- *Funding*: In this project, resources in form of field guides and instruction by scientists are provided, and observation sites are usually located close to schools or neighborhoods, removing some of the financial costs and issues with accessibility/ facilities that may be cost prohibitive for participants in other instances (Hobbs & White, 2012; Saunders et al., 2018; Turrini et al., 2018). However, some Norwegian and Finnish schools dropped out because there was not enough funding for traveling to teacher and pupil meetings. The project provides now some funding for Norwegian schools when the meetings are in Russia to increase long‐term participation. Funding mainly comes from the Norwegian government, and the initial funding has been for Norwegian and Russian schools, and therefore, no such financial support can be given to Finnish schools at the moment. Hence, additional funding sources need to be secured to increase the involvement of Finnish schools, which have experienced financial cuts for participation in such projects in recent years. More generally, funding agencies for either research institutes/state nature reserves or schools should consider funding these types of costs to enable continuous teacher and pupil education, data generation, and project‐based learning experiences. Our experiences are in line with other assessments that show that staff shortages and insecure funding in addition to time constraints represent major barriers to citizen science project implementation (Turrini et al., 2018).
- *Insufficient teacher training*: Citizen science projects often emphasize different pedagogies and learning outcomes (Table 2; Cebrián & Junyent, 2015; DITOs Consortium, 2019; Ruiz‐Mallén et al., 2016; Wals et al., 2014). However, some teachers have had difficulties with the implementation of the project‐based work suggesting that more support for teachers is needed to bring this type of educational tool into schools. Planning time, flexibility in curriculum implementation, and access to resources like lesson plans have been proposed as key factors for successful project‐based learning (DITOs Consortium, 2019). Recently, Norway has implemented a new national curriculum (http://www.udir.no/kl06/ENG01-04) that puts more emphasis on knowledge democratization, active participation of pupils in learning activities, learning in an interdisciplinary setting, and sustainable development. Therefore, we are optimistic that this project will soon be more interesting again for Norwegian schools.
- *Teachers’ unfamiliarity with environmental education in a multidisciplinary context*: Another challenge has been the implementation of multidisciplinary projects parts that dealt with environmental education rather than knowledge and practical skills. For example, art has been proposed as an effective way to teach environmental education (e.g., Fragkoulis & Koutsoukos, 2018). However, mainly biology teachers were interested in this project and teachers with other backgrounds have had problems with connecting the project to their subject. This suggests that some teachers need further education in multi‐ and interdisciplinary pedagogics.
- Another possibility is that there has been a bias in perceived importance of different aspects so that knowledge components like direct knowledge of the natural environment (e.g., species) are taught by teachers more intensively and probably these aspects fit better with existing school curricula than learning components connected to attitudes and emotions. In a survey asking primary teacher students about main learning outcomes in scientific and environmental education, a bias in perceived importance of different knowledge types, with ethical values, attitudes, and emotions scoring far lower than knowledge and practical skills, was found (Cebrián & Junyent, 2015). Although this should be less of an issue in middle school, our experience has been that national school curricula do not necessarily permit for this type of learning and/or some teachers have been unfamiliar or uncomfortable to teach in a different way they are used to. Although the established scientists–school partnerships alleviate this problem to some extent, more work in this direction remains to be done and will require funding from governmental agencies along with institutional changes to enable scientists and teachers spending time on these activities, creating continuous learning opportunities for school teachers. Funding schemes need to be flexible enough so that different items (e.g., travel costs, school equipment like computers) can be applied for by different actors (e.g., teachers and scientists) to address different needs in different schools and countries depending on local conditions and facilities (DITOs Consortium, 2019; Turrini et al., 2018).

## 8 RECOMMENDATIONS

Based on our experiences with this long‐term program, we advise to consider the following points, when thinking about starting a similar initiative:

- Discuss with schools their long‐term commitment to the program beforehand, probably at the management level rather than at the individual teacher level, to achieve integration of program into school curriculum as much as possible.
- Before discussing with schools, prepare clear notes what the program contributes to the school curriculum and which learning outcomes can be expected. Tables 1 and 2 of this article may serve as inspiration to showcase potential learning outcomes. This is an important step as teachers will be reluctant to join the project if the benefits of participation are unclear.
- If integration into the school curriculum is not feasible, can the program run as an extracurricular activity (e.g., phenology club)? Although this option will have a much‐reduced audience, in some instances, it may be a better option to at least reach some pupils. However, careful planning is necessary to keep these initiatives running as well.
- Consider developing teaching tool kits as an additional help provided free of charge to teachers that are available online. These could include short videos explaining biological and technical aspects of the project as well as teaching materials on how to set up a multidisciplinary project like this in schools, including exercises and games in different school subjects (e.g., biology, art, mathematics/statistics, politics, social sciences, geography, language courses). Again, special attention should be paid to explaining to teachers how the program will achieve certain learning outcomes.
- Think about the long‐term goals of the project and potential extensions. For example, involving former pupils, now adults, could extent the program beyond a school context. Involvement of current and former pupils would help to transition the project into a community science project that is carried out by different societal groups to facilitate local and regional monitoring projects and has the potential of more active participation in form of co‐developing local research questions, collecting and analyzing data as well as dissemination of results (Harris et al., 2020; Shirk et al., 2012).

Finally, one important recent development is that translation of the website into the local native Sámi language has begun. This addresses the previously largely neglected inclusion of native northern communities in this activity for mutual knowledge exchange. One important lesson learned here is that teaching and research materials are often not readily available in indigenous languages, which hinders knowledge exchange and involvement of native communities in the coproduction of locally relevant knowledge for sustainable development. The inclusion of indigenous communities will aid in the reduction of inequalities and increased cross‐cultural understanding (United Nations Department of Public Information, 2019). Indigenous knowledge can also serve as legacy data and baseline data to extend the existing data set and knowledge network. Lastly, even small microclimatic changes can result in changes in subsistence practices (McNeeley & Shulski, 2011). Thus, the inclusion of traditional ecological knowledge holders may provide further insight into regional socio‐economic changes, further encouraging the development of a community science program in the long term.

## CONFLICT OF INTEREST

The authors declare no competing or financial interests.

## AUTHOR CONTRIBUTIONS

**Cornelya F. C. Klűtsch:** Conceptualization (equal); writing – original draft (lead); writing – review and editing (lead). **Paul Eric Aspholm:** Conceptualization (equal); investigation (equal); writing – review and editing (equal). **Natalia Polikarpova:** Conceptualization (equal); investigation (equal); writing – review and editing (equal). **Olga Veisblium:** Conceptualization (equal); investigation (equal); writing – review and editing (equal). **Tor‐Arne Bjørn:** Investigation (equal); project administration (equal); writing – review and editing (equal). **Anne Wikan:** Project administration (equal); writing – review and editing (equal). **Victoria Gonzalez:** Investigation (equal); methodology (equal); writing – review and editing (equal). **Snorre B. Hagen:** Project administration (equal); supervision (lead); writing – review and editing (equal).
